# Influence of Surface Contaminants and Hydrocarbon Pellicle on the Results of Wettability Measurements of Titanium

**DOI:** 10.3390/ijms241914688

**Published:** 2023-09-28

**Authors:** Daisuke Kido, Keiji Komatsu, Toshikatsu Suzumura, Takanori Matsuura, James Cheng, Jeong Kim, Wonhee Park, Takahiro Ogawa

**Affiliations:** 1 Weintraub Center for Reconstructive Biotechnology, Division of Regenerative and Reconstructive Sciences, UCLA School of Dentistry, Los Angeles, CA 90095-1668, USA; 2 Department of Oral Diagnosis and General Dentistry, Graduate School of Medical and Dental Sciences, Tokyo Medical and Dental University (TMDU), 1-5-45 Yushima, Bunkyo-ku, Tokyo 113-8510, Japan

**Keywords:** titanium implants, wettability, osseointegration, bone integration, UV photofunctionalization

## Abstract

Hydrophilicity/hydrophobicity—or wettability—is a key surface characterization metric for titanium used in dental and orthopedic implants. However, the effects of hydrophilicity/hydrophobicity on biological capability remain uncertain, and the relationships between surface wettability and other surface parameters, such as topography and chemistry, are poorly understood. The objective of this study was to identify determinants of surface wettability of titanium and establish the reliability and validity of the assessment. Wettability was evaluated as the contact angle of ddH_2_O. The age of titanium specimens significantly affected the contact angle, with acid-etched, microrough titanium surfaces becoming superhydrophilic immediately after surface processing, hydrophobic after 7 days, and hydrorepellent after 90 days. Similar age-related loss of hydrophilicity was also confirmed on sandblasted supra-micron rough surfaces so, regardless of surface topography, titanium surfaces eventually become hydrophobic or hydrorepellent with time. On age-standardized titanium, surface roughness increased the contact angle and hydrophobicity. UV treatment of titanium regenerated the superhydrophilicity regardless of age or surface roughness, with rougher surfaces becoming more superhydrophilic than machined surfaces after UV treatment. Conditioning titanium surfaces by autoclaving increased the hydrophobicity of already-hydrophobic surfaces, whereas conditioning with 70% alcohol and hydrating with water or saline attenuated pre-existing hydrophobicity. Conversely, when titanium surfaces were superhydrophilic like UV-treated ones, autoclaving and alcohol cleaning turned the surfaces hydrorepellent and hydrophobic, respectively. UV treatment recovered hydrophilicity without exception. In conclusion, surface roughness accentuates existing wettability and can either increase or decrease the contact angle. Titanium must be age-standardized when evaluating surface wettability. Surface conditioning techniques significantly but unpredictably affect existing wettability. These implied that titanium wettability is significantly influenced by the hydrocarbon pellicle and other contaminants inevitably accumulated. UV treatment may be an effective strategy to standardize wettability by making all titanium surfaces superhydrophilic, thereby allowing the characterization of individual surface topography and chemistry parameters in future studies.

## 1. Introduction

The hydrophilic or hydrophobic state (or wettability) of titanium is a major surface characterization metric in studies of titanium and implant materials [1,2,3,4,5,6,7,8,9,10,11,12,13,14], and it is commonly assessed by measuring the contact angle of water [15,16,17,18,19,20,21,22,23]. Although the definition varies and is used differently in different fields, representative definitions are superhydrophilic 0° < θ < 10°, hydrophilic 10° < θ < 30°, hydrophobic 30° < θ < 90°, and hydrorepellent θ > 90° [23,24,25]. In clinical and biological studies of dental and orthopedic titanium implants, the hydrophilicity/hydrophobicity of implant surfaces is considered a critical factor that influences the biological capability–specifically osseointegration–of implants [26,27,28,29,30,31,32,33,34,35,36,37,38].

However, the exact relationship between surface wettability and osseointegration remains controversial, for several possible reasons. First, the hydrophilicity/hydrophobicity of most titanium surfaces is relatively constant, ranging from 60° to 120° [16,39,40,41,42,43], i.e., hydrophobic or hydrorepellent, making it difficult to establish strong correlations with biological parameters. Second, it is technically challenging to produce a continuous range of hydrophilicity/hydrophobicity on experimental specimens; UV treatment of titanium surfaces makes them superhydrophilic (contact angle of 0° or <10° for most titanium surfaces, regardless of surface topography) [17,44,45,46], and most titanium surfaces are superhydrophilic or hydrophilic immediately after surface processing [15,42,44], so titanium surfaces tend to lie at the extreme ends of the wettability spectrum. Lastly and more importantly, the reliability and validity of contact angle measurement have not been established, i.e., the surface factors and measurement conditions that influence wettability have not been fully defined [47,48,49]. Systematic analyses of contact angles that consider surface topography, surface conditioning, measurement protocol, titanium age, and their synergy are urgently required.

Therefore, the objective of this study was to determine the reliability and validity of hydrophilicity/hydrophobicity measurements of titanium surfaces by examining the independent and combined effects of surface topography, titanium age, measurement protocol, and surface conditioning on the contact angle of water. Establishing the critical determinants of hydrophilicity/hydrophobicity is expected to improve the design and interpretation of future studies of titanium and implant materials.

## 2. Results

### 2.1. Surface Characteristics of Titanium Specimens

**Figure 1:**
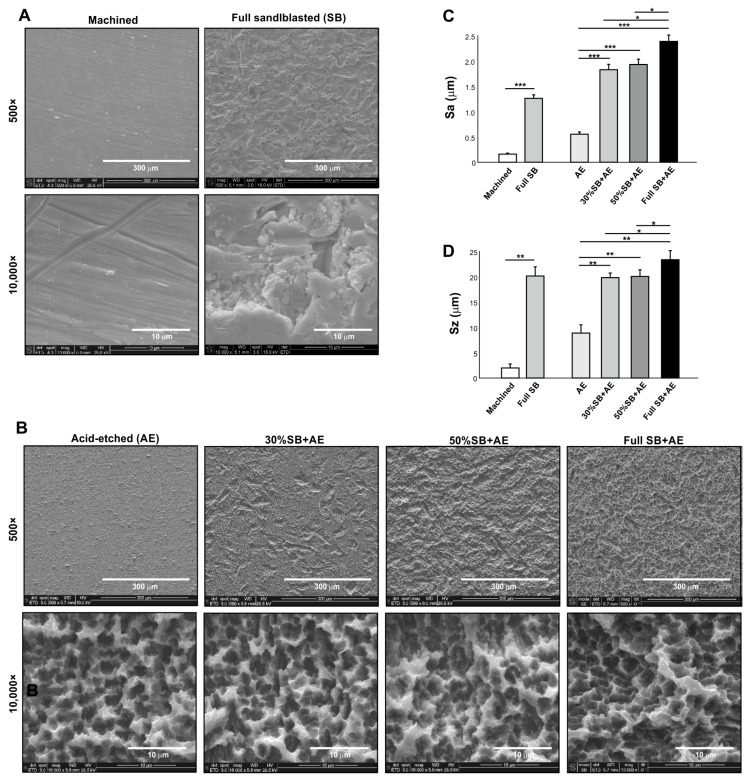
Surface characterization of titanium specimens used in this study. Scanning electron microscopy (SEM) images (**A**,**B**), average roughness (Sa) (**C**), and peak-to-valley roughness (Sz) (**D**) of the specimen surfaces: * *p* < 0.05, ** *p* < 0.01, *** *p* < 0.001.

SEM images of machined titanium surfaces showed no defined morphology except for the scratches and traces from machine milling (Figure 1A). Sandblasted surfaces showed relatively larger-scale roughness (range 10–20 mm) and random morphology. All acid-etched titanium surfaces showed microscale roughness consisting of peaks and pits in high-magnification images (Figure 1B). Depending on the duration of sandblasting, there was progressive formation of supra-micron larger scale roughness, with the full SB + AE surface showing the densest crater-like supra-micron concavities.

Quantitative roughness analysis showed the lowest average roughness (Sa) for machined surfaces and the highest Sa for full SB + AE surfaces (Figure 1C). A combination of sandblasting and acid-etching (SB + AE surfaces) effectively increased the Sa. Peak-to-valley roughness (Sz) results were similar, with the full SB + AE surfaces being the highest and the machined surfaces being the lowest (Figure 1D). The exception was that the full SB surfaces showed an equivalent Sz to those on SB + AE surfaces.

### 2.2. Effect of Aging on Hydrophilicity/Hydrophobicity of Titanium with Different Topographies

**Figure 2:**
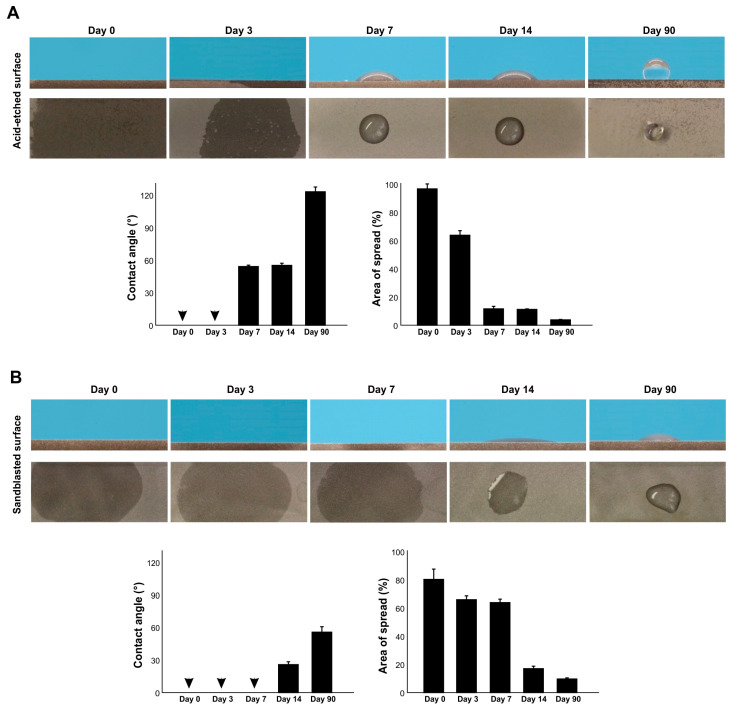
The effect of age on the hydrophilicity/hydrophobicity of titanium. Hydrophilicity/hydrophobicity of differently aged specimens was evaluated by the contact angle and the area of spread of 3 µL ddH_2_O placed on titanium specimens. Side- and top-view photographs of a water droplet and histograms are presented. (**A**) Acid-etched titanium specimens. (**B**) Sandblasted titanium specimens. Arrowheads indicate 0°.

We next examined the surface wettability of titanium surfaces over time since surface processing by measuring the contact angle of 3 µL ddH_2_O on acid-etched and sandblasted surfaces. Both acid-etched and sandblasted surfaces were superhydrophilic immediately after surface processing, with a contact angle of 0° (Figure 2A,B). The contact angle remained at 0° 3 days later for both surfaces. However, after 7 days, the contact angle of acid-etched surfaces was over 50°, whereas sandblasted surfaces still had a contact angle of 0°. The contact angle increased over time for both surfaces, rapidly so for acid-etched surfaces. The acid-etched and sandblasted surfaces were hydrorepellent and hydrophobic, respectively, after 90 days.

We also measured the area of water spread, which was more sensitive to titanium age than the contact angle (Figure 2A,B). The area of spread was larger for acid-etched surfaces than sandblasted surfaces immediately after surface processing and started to shrink on day 3. The rate of decrease in spread area was faster for acid-etched surfaces. Although the contact angle on sandblasted surfaces remained at 0° until day 7, the area of spread steadily decreased over this time. Thus, there was age-related degradation of hydrophilicity, and the rate was topography-specific.

### 2.3. Inter- and Intra-Specimen Reliability and Intra-Droplet Reliability of Contact Angle Measurements

**Figure 3:**
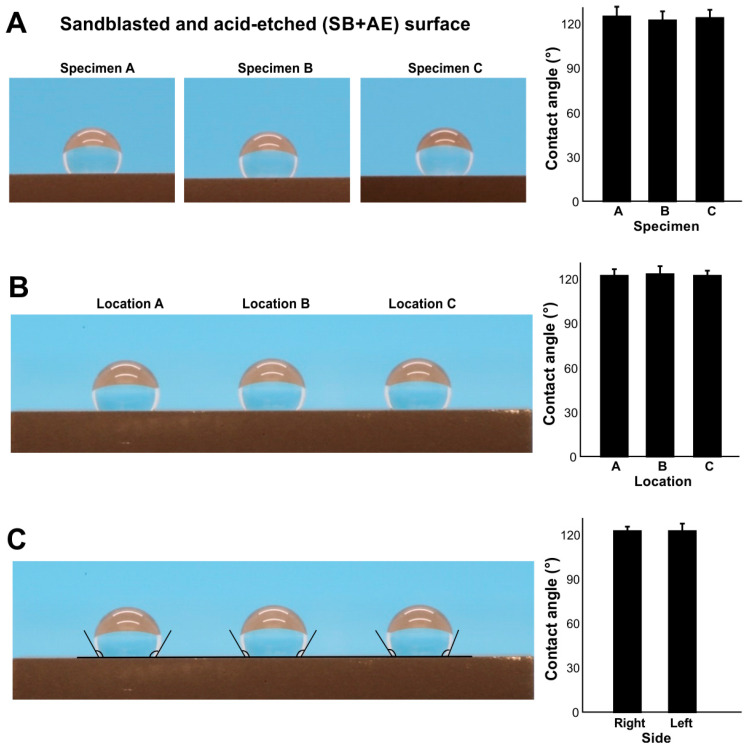
The reliability of contact angle measurement. To standardize the age of titanium, all specimens were stored for 90 days. (**A**) Inter-specimen reliability evaluated on sandblasted and acid-etched (SE + AE) titanium specimens. Side-view photographs of 3 µL of ddH_2_O placed on the three different specimens and the calculated contact angles. (**B**) Intra-specimen reliability. Three drops of 3 µL ddH_2_O placed in three different zones on the same specimen. (**C**) Intra-droplet reliability. The contact angle was measured at both sides of 3 µL ddH_2_O droplets.

We next examined the inter- and intra-specimen stability of contact angle measurements using full SB + AE surfaces aged to 90 days. There was no significant difference in contact angle between three different specimens (Figure 3A) nor at three areas within an individual specimen (Figure 3B). The contact angle was the same on the right and left sides of the water droplets (Figure 3C). Therefore, at least for age-standardized titanium at 90 days, contact angle measurements showed intra- and inter-specimen reliability.

### 2.4. Effect of Water Volume on Contact Angle

**Figure 4:**
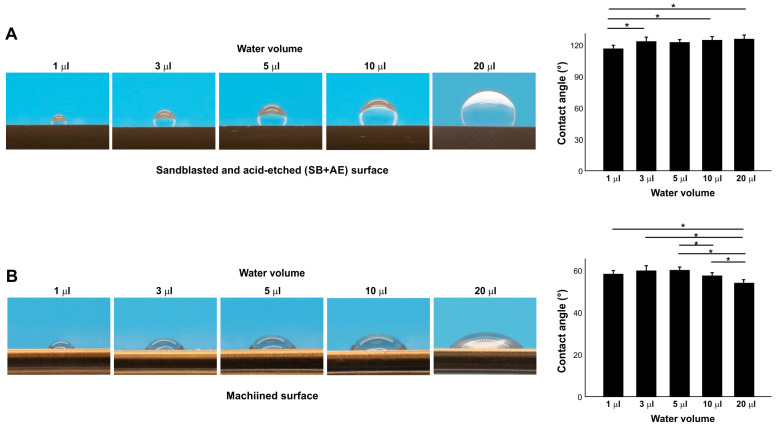
The effect of water volume on contact angle measurement on sandblasted and acid-etched (SB + AE) (**A**) and machined (**B**) surfaces. The specimens were used 90 days after surface processing: * *p* < 0.05.

We next examined the effect of different water volumes on contact angle (Figure 4A,B) on two different surfaces: full SB + AE and machined surfaces. On the full SB + AE surfaces, the contact angle increased from 1 µL to 3 µL but was then unchanged up to 20 µL (Figure 4A). On the machined surface, the contact angle was unchanged up to 5 µL but decreased with larger volumes (Figure 4A), revealing a surface topography-specific bias in measurement according to water volume.

### 2.5. Effect of Surface Roughness on Contact Angle

**Figure 5:**
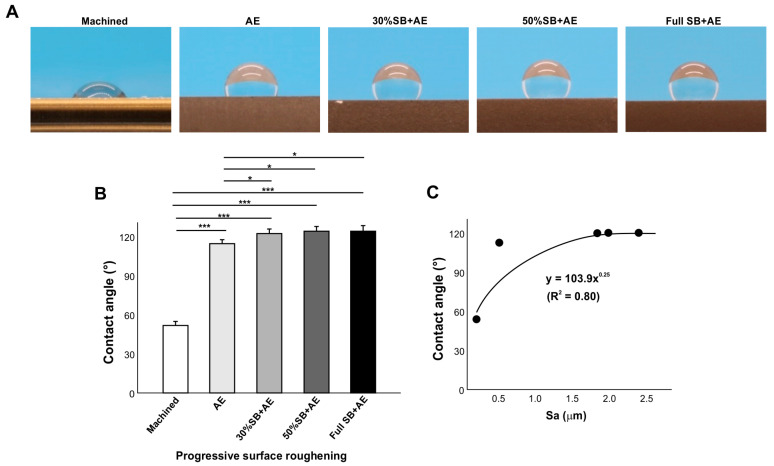
The effect of surface roughness on contact angle. Machined surfaces and acid-etched surfaces pre-sandblasted for various times were tested. The specimens were used 90 days after surface processing. Side-view photographs of 3 µL ddH_2_O (**A**) and the calculated contact angles (**B**). The plot of contact angle against the average surface roughness (Sa) of each of the variously roughened titanium surfaces, with an exponential correlation curve (**C**): * *p* < 0.05, *** *p* < 0.001.

We compared the contact angle on acid-etched titanium surfaces of different roughness by altering the sandblasting time (Figure 5). The contact angle was significantly greater on the AE surface than on the machined surface and further increased on the 30% SB + AE surface (Figure 5A,B). However, further increases in sandblasting time did not further increase the contact angle, which plateaued. The regression analysis of the contact angle and Sa showed a significant exponential correlation, indicating a positive trend between surface roughness and the contact angle (Figure 5C).

### 2.6. Effect of Surface Roughness on UV-Treated Surfaces

**Figure 6:**
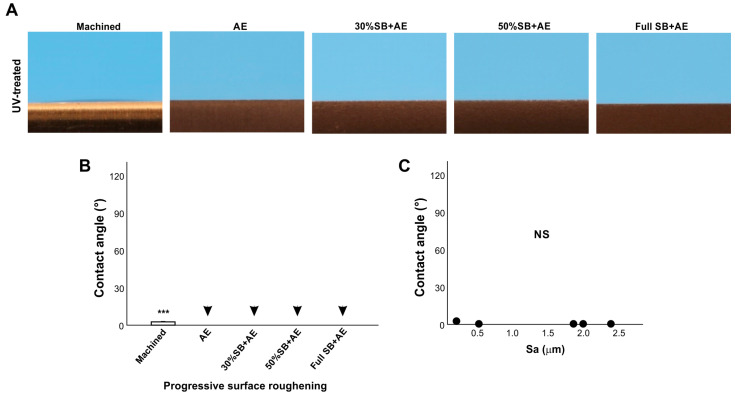
The effect of surface roughness on contact angle on UV-treated titanium surfaces. Machined surfaces and acid-etched surfaces pre-sandblasted for various times were stored for 90 days and treated with UV light prior to testing. Side-view photographs of 3 µL ddH_2_O (**A**) and the calculated contact angles (**B**). The plot of the contact angle against the average surface roughness (Sa) of each of the various roughened titanium surfaces (**C**). There were no statistically significant correlations between parameters. Arrowheads indicate 0°: *** *p* < 0.001. NS: not significant.

We next examined the effect of surface roughness on UV-treated titanium surfaces using the same specimens as in Figure 6 treated with VUV light for one minute (Figure 6). The contact angle of the machined smooth surface decreased from ~60° to less than 10°, indicating that the surface had become superhydrophilic (Figure 6A,B). Regardless of roughness, i.e., different durations of sandblasting, all acid-etched surfaces became superhydrophilic after UV treatment, with a contact angle of 0°, so there was no significant correlation in regression analysis and the trend in Figure 6 was rather opposite (Figure 6C).

### 2.7. Effect of Surface Conditioning on Contact Angle

**Figure 7:**
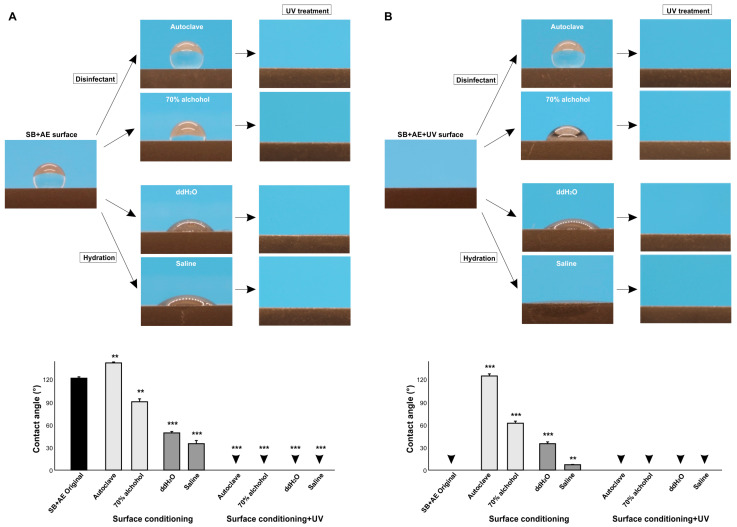
The effect of surface conditioning on the contact angle. The 90-day-old sandblasted and acid-etched (SB + AE) surfaces (**A**) and those with UV treatment (**B**) were tested. The specimens were conditioned using four different techniques. Side-view photographs of 3 µL ddH_2_O and the calculated contact angles are shown. Arrowheads indicate 0°: ** *p* < 0.01, *** *p* < 0.001.

We next examined the effect of various surface conditioning techniques on the contact angle. To model disinfectants used clinically and experimentally, we examined autoclaving and cleansing with 70% alcohol, while to model clinically applicable hydration, we soaked titanium specimens in ddH_2_O or saline. Standard 90-day-old SB + AE specimens were tested. Compared with the baseline hydrorepellent state, autoclaving further increased the contact angle and significantly increased hydrophobicity, whereas alcohol cleansing decreased the contact angle (Figure 7A). Hydrating the specimens significantly decreased the contact angle, with saline having a greater effect than ddH_2_O. However, the contact angle remained >30° and outside the range of hydrophilicity.

We next carried out the same experiment using UV-treated specimens (Figure 7B). UV-treated SB + AE surfaces were superhydrophilic with a 0° contact angle, but both disinfecting techniques reversed the wettability from superhydrophilic to hydrophobic, with autoclaving having a greater effect than alcohol cleaning and turning the specimens hydrorepellent. Hydration also reduced UV-generated superhydrophilicity, with the contact angle increasing more with ddH_2_O than with saline.

### 2.8. Generation and Recovery of Superhydrophilicity via UV Treatment

Given that surface conditioning increased hydrophobicity, we next determined if UV treatment overcame this effect. Regardless of pre-UV treatment, treatment with UV light made all surfaces superhydrophilic without exception (Figure 7A,B), indicating that superhydrophilicity can be newly generated even after surface conditioning and that superhydrophilicity can be recovered even on surfaces compromised by surface conditioning.

**Figure 8:**
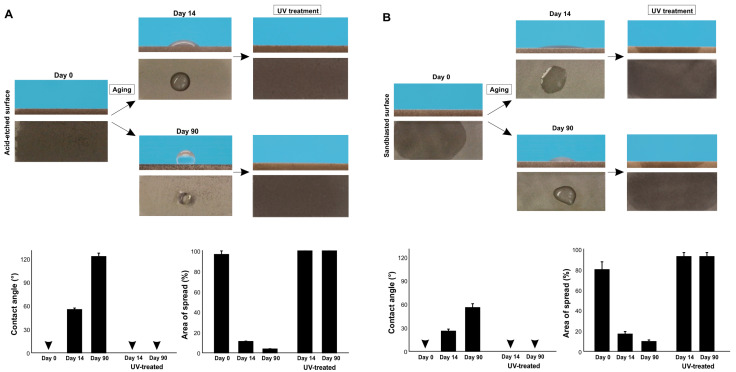
Restoration of superhydrophilicity via UV treatment. Differently aged titanium surfaces after acid-etching (**A**) and sandblasting (**B**) were treated with VUV light for one minute. Side- and top-view photographs of 3 µL ddH_2_O and the calculated contact angles and the area of water spread are shown. Arrowheads indicate 0°.

Finally, we determined if UV treatment can regenerate superhydrophilicity on differently aged surfaces (Figure 8A,B). Regardless of acid-etching or sandblasting, 14-day-old and 90-day-old surfaces became superhydrophilic with a 0° contact angle after UV treatment. The water spread area was even greater after UV treatment than on day 0 titanium specimens, indicating that the degree of superhydrophilicity increased further by UV treatment compared with the state of new surfaces.

## 3. Discussion

**Figure 9:**
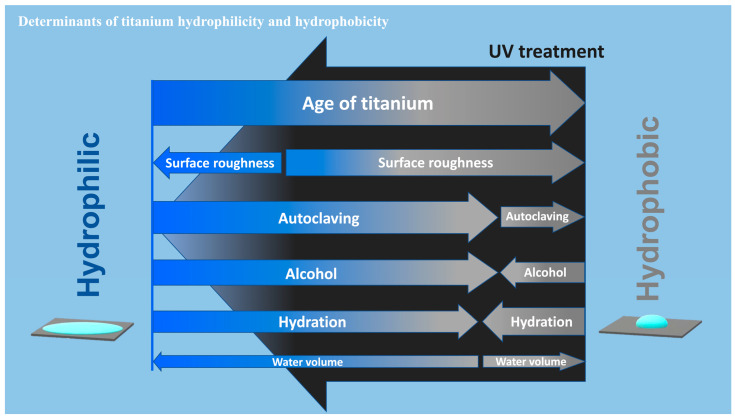
A diagram showing the determinants of hydrophilic/hydrophobic property or wettability of titanium surfaces. The widths of arrows represent the degree of impact of each factor on the water contact angle. Note that the impact toward hydrophilic or hydrophobic trend may not be mono-directional for some factors depending on the existing wettability. UV treatment turns titanium surfaces superhydrophilic, regardless of the degree or involvement of all factors tested.

We established that some factors could have profound effects on the wettability of titanium, in some cases from superhydrophilic to hydrorepellent or vice versa. Highlights of the present results are summarized in a diagram (Figure 9). The most significant factor was the age of the titanium, with the contact angle significantly increasing (markedly reduced hydrophilicity) as the specimen aged after surface processing. This age-driven loss of hydrophilicity was previously reported for acid-etched and machined titanium surfaces [9,41,42], and is known as the “biological aging” of titanium [9]. Here we found that sandblasted surfaces also undergo biological aging, although the aging was significantly slower on sandblasted surfaces than on acid-etched surfaces. Sandblasted surfaces maintained superhydrophilicity up to day seven and still did not show hydro-repellency even after 90 days. These results suggest a combinational effect of surface aging and topography in determining titanium wettability and that age or topography alone cannot predict the contact angle. Similar aging rate modulation was observed on titanium dioxide-coated and nano-structured titanium [44]. Another study reported faster aging, i.e., degradation of hydrophilicity on rougher titanium surfaces when titanium specimens were submerged in saliva [50]. There seem to be ways to delay aging but no way to prevent it [9,51]. Furthermore, regardless of surface topography, titanium surfaces were superhydrophilic immediately after surface processing. This was surprising, since titanium surfaces are traditionally considered hydrophobic [52,53,54,55], but our data suggest that this is probably because the titanium specimens and implant products used in previous reports were sufficiently old. The physicochemical explanation for age-related loss of hydrophilicity is the pellicle of hydrocarbon molecules that naturally and inevitably form on titanium surfaces over time [9,16,41,56,57]. A few studies reported hydrophilic titanium surfaces after particular surface modification without mentioning the age of those specimens [58,59], which needs careful interpretation to preclude the possibility that they were hydrophilic just because they are new after processing. An attempt was made to prevent the pellicle accumulation by packaging titanium implants in the saline solution while handing under nitrogen inactive gas [43]. However, carbon elements were detected on these implants [43,60]. Soaking titanium in the saline solution or water did not prevent pellicle accumulation, resulting in the degradation of the bioactivity of the titanium [61]. Implant products, as medical devices, are commonly packaged and sealed individually. However, a wide variety of surface carbon ranging from 25 to 76% is detected on these products [62,63] and the surfaces are hydrophobic [38,64].

Few studies have explored the effect of surface roughness on the wettability of titanium. We found that the rougher the surface, the more hydrophobic it became (Figure 5), with the machined surface the less hydrophobic. However, the effect of surface roughness was not linear and there was a significant exponential correlation, indicating a limit to the effect of surface roughness. The contact angle plateaued at ~120° when the average surface roughness was 1.88 mm on 30% SB + AE surfaces, and further roughening did not negatively impact wettability. A study compared the contact angle on titanium surfaces that were acid-etched at various temperatures [55]. It was shown that the contact angle was higher on titanium specimens etched at higher temperature. Since high-temperature acid-etching creates rougher surfaces, the result supported the general trend found in the present study that the rougher the surface, the more hydrophobic.

Of note, the positive correlation between surface roughness and hydrophobicity did not apply to UV-treated titanium surfaces. UV treatment converted all surfaces to superhydrophilic. Indeed, UV treatment is known to turn titanium surfaces hydrophilic by removing the hydrocarbon pellicle [10,24,27,28,29,61]. After UV treatment, the effect of surface roughness was nearly completely negated, and there was no correlation between the average surface roughness and the contact angle. Rougher surfaces, when aged, plausibly contain more hydrocarbon due to their larger surface area. This could be an explanation for why rougher surfaces are more hydrophobic. Conversely, when the rougher surfaces are free from hydrocarbon after UV treatment, they are superhydrophilic regardless of the degree of roughness. Although all surfaces tested were superhydrophilic after UV treatment, the contact angle was highest on machined surfaces, opposite to the principle that the rougher the surface, the more hydrophobic it becomes. Thus, UV treatment creates a special and distinct phenomenon of surface wettability. We propose that, regardless of UV treatment, surface roughness accentuates the wettability determined by the existing physicochemical properties of the titanium. For instance, regular “old” titanium surfaces are hydrophobic due to the hydrocarbon pellicle and become more hydrophobic when the surfaces are rougher. Conversely, UV-treated surfaces are hydrophilic due to their pellicle-free surface and become more hydrophilic when the surfaces are rougher.

Of the four surface conditioning techniques tested, autoclaving and alcohol cleaning, which are standard ways to disinfect specimens and devices in routine clinical and experimental settings, significantly affected the wettability of aged titanium surfaces. The two techniques had opposite effects, with autoclaving enhancing hydrophobicity and alcohol cleaning attenuating hydrophobicity, a finding that requires the assessment of other physicochemical factors that might be altered by these two techniques, including the highly sensitive elemental and isotopic analysis via X-ray photoelectron spectroscopy (XPS) [65,66] and laser ablation–inductively coupled plasma–mass spectrometry (LA-ICP-MS) [67,68,69]. Variations of the contact angle presumably by different cleaning techniques is reported by other studies [49,70] but no principle or mechanism was established. Autoclaving might promote carbon accumulation on titanium [71]. The autoclave used in this study was typical for clinical and research applications, but there are minor variations in steam impurities, pH, drying, remnant detergents, and other unknown factors among the devices [72]. It seems extremely difficult to control the conditions and prevent accidental contamination. Further research is needed to generalize the effects of these disinfecting techniques and identify the determinants for altered wettability. Hydration was expected to alleviate the hydrophobicity of aged titanium surfaces due to the deposition of water molecules or hydroxyl groups on the surfaces [2,73], which was supported by the results. The greater effect seen with saline compared with ddH_2_O implied that the ionic interactions between saline and titanium promoted a hydrophilic state. All of the results were very different when the four conditioning techniques were applied to pre-existing (UV-treated) superhydrophilic titanium surfaces, with the contact angle remarkably increasing from 0° regardless of the conditioning technique in most cases. Superhydrophilicity was completely abolished by autoclaving, alcohol cleaning, and soaking in ddH_2_O, with the greatest negative impact seen with autoclaving. Soaking in saline maintained superhydrophilicity, probably due to the positive effects of the ionic interactions. However, even with saline, UV-induced superhydrophilicity was significantly compromised.

Inter- and intra-specimen reproducibility analysis suggested that, provided that the surface processing undergoes quality control and the age of specimens is standardized, measuring the contact angle of water is a reliable means to evaluate the hydrophilicity or hydrophobicity of titanium surfaces. The effect of water volume was significant and specific; plausibly due to gravity, a greater volume of water increased the contact angle on hydrorepellent surfaces but decreased it on hydrophobic surfaces. This finding has important implications, in that absolute contact angles cannot be compared between different studies unless the water volume is standardized. Indeed, the reduced reliability of contact angle measurement was reported with the use of water of 30 mm or more [49]. On the other hand, a very small volume may introduce possible technical error. To minimize the effect of gravity and technical error, this study used a 3 µL protocol.

Our results also highlight issues in the evaluation of surface wettability across many biomedical, engineering, and clinical domains, since wettability has been used extensively in these studies as a key surface characterization metric to interpret the results and infer mechanisms associated with surface topography and chemistry. For instance, with respect to titanium age, our findings make it clear that comparing the hydrophilicity or hydrophobicity between different experimental specimens is meaningless unless they are standardized for age, and even then surface processing and topography must be considered since the rate of aging significantly differs between acid-etched and sandblasted surfaces. Similarly, surface conditioning impacted the results; regardless of experimental or clinical use, titanium specimens and devices require final conditioning prior to use, and different studies use different disinfecting techniques and rinsing protocols, the specifics of which are often omitted from the published experimental protocol. Future studies should detail the conditioning protocol.

The biological impact of the high surface energy of titanium and other biomaterials was extensively reported. High-energy titanium surfaces, represented by superhydrophilicity, recruit more osteoblasts and promote osseointegration [9,13,16,41,42,44]. The high-energy titanium surfaces created by UV treatment or UV photofunctionalization are also studied extensively for their ability to enhance osseointegration and soft tissue responses at the cell [12,74], animal [74,75,76], and clinical [28,77] levels. The high surface energy is created by the UV-mediated removal of the hydrocarbon pellicle via three different mechanisms (1) photochemical decomposition (ozone-mediated or non-mediated cleaning); (2) photophysical decomposition (direct bond dissociation by UV energy); and (3) photocatalytic decomposition (UV-titanium interaction) [78,79].The present study proposes another benefit of UV treatment, namely to standardize the wettability of titanium, because UV treatment converted all titanium specimens with different surface topographies, ages, and conditionings to superhydrophilic, allowing future studies to focus on the effect of other specific surface parameters such as topography and chemistry. As discussed above, the accumulation of the hydrocarbon pellicle and other chemical contaminants is nearly inevitable and there is no method to prevent the time-related loss of hydrophilicity. As shown by the rejuvenation of the aged surface, UV treatment produced even higher superhydrophilicity than new surfaces. Furthermore, with the use of high-energy VUV light, superhydrophilic standardization can be accomplished in a minute, minimally impacting the experimental protocol [78,79]. This study did not study every surface type used in the field, and further studies of other surfaces with nano- and Meso-level topography and other biological and chemical modifications and coatings are now warranted.

## 4. Materials and Methods

### 4.1. Titanium Specimens and Surface Processing

Titanium specimens in rectangular plate form (14 × 6 × 2 mm) were prepared by machine-milling grade IV commercially pure titanium. To create surface topography, specimens were modified by sandblasting, acid-etching, or a combination of both. Sandblasting (SB) was carried out with Al_2_O_3_ particles (70 mesh) for either 1.65 (30% SB), 2.75 (50% SB), or 5.5 (full SB) seconds. Acid-etching was performed by processing specimens in HCl and H_2_SO_4_ at 95 °C for 6 min and 30 s. The specimens were prepared and provided by DIO Implant (Busan, Republic of Korea) and individually packaged and sealed in a quartz ampoule. For aging studies, specimens were stored in dark, ambient conditions in a sealed package at 25 °C up to 90 days.

### 4.2. Surface Conditioning and UV Treatment

Four different surface conditioning techniques were used: (i) surface disinfecting by autoclaving at 2.0 bar at 121 °C for 20 min, followed by 10 min drying in a sealed sterilization pouch (Fisher Scientific, Pittsburgh, PA); (ii) cleaning by immersing specimens in 70% ethanol for 24 h followed by rinsing in ddH_2_O for 20 min and drying; hydrating by immersing specimens in (iii) ddH_2_O or (iv) saline solution for 24 h and then drying. UV treatment was performed at room temperature using a vacuum UV (VUV) light (172 nm vacuum UV, 60 mW/cm^2^) (DIO Implant, Busan, Republic of Korea) for 1 min [71,72].

### 4.3. Surface Characterization and Wettability Testing

The surface morphology of titanium specimens was qualitatively examined by scanning electron microscopy (SEM; Nova 230 Nano SEM, FEI, Hillsboro, OR, USA). In addition, roughness was quantified using an optical profile microscope (MeX, Alicona Imaging GmbH, Raaba, Graz, Austria) to measure the average roughness (Sa) and peak-to-valley roughness (Sz). The hydrophilicity/hydrophobicity or wettability of specimen surfaces was evaluated by measuring the contact angle of 3 µL of ddH_2_O in most experiments. To examine the effect of water volume, the contact angle was also measured with 1, 5, 10, and 20 µL ddH_2_O.

### 4.4. Statistical Analysis

Contact angle measurements were performed on three independent titanium specimens in each group in each experiment. The effects of titanium age, different droplets, surface roughness, water volume, surface conditioning, and UV treatment were compared by one-way ANOVA. Bonferroni’s test was used as a post hoc multiple comparison test where appropriate. *p*-values < 0.05 were considered statistically significant. Regression analysis was applied to determine associations between the average surface roughness and the contact angle.

## 5. Conclusions

This study identified important factors that determine the hydrophilicity/hydrophobicity (or wettability) of titanium surfaces. The effect of titanium age was critical and decreased the contact angle from superhydrophilic to hydrorepellent depending on age and topography. Different surface conditioning techniques also altered the wettability, and the alterations were diverse and specific to the existing wettability state; for instance, UV-treated superhydrophilic surfaces became hydrorepellent after autoclaving. These implied that titanium wettability is significantly influenced by the hydrocarbon pellicle and other contaminants inevitably accumulated. Our study highlights that hydrophilicity/hydrophobicity assessment and interpretation require careful consideration of measurement approach, handling, and conditioning. UV treatment may be an effective, novel strategy to standardize the wettability of titanium by making all surfaces superhydrophilic and allowing assessment of other individual factors including but not limited to surface topography and chemistry.

## Data Availability

The data presented in this study are available upon request from the corresponding author.
